# Effect of anemoside B4 on milk whey in clinical mastitis-affected cows elucidated using tandem mass tag (TMT)-based quantitative proteomics

**DOI:** 10.1038/s41598-022-23749-x

**Published:** 2022-11-05

**Authors:** Liu-hong Shen, Yue Zhang, Yu Shen, Zhe-tong Su, Shu-min Yu, Sui-zhong Cao, Xiao-lan Zong

**Affiliations:** 1 The Key Laboratory of Animal Disease and Human Health of Sichuan Province, The Medical Research Center for Cow Disease, College of Veterinary Medicine, Sichuan Agricultural University, Chengdu, 611130 Sichuan China; 2 Guangxi Innovates Medical Technology Co., Ltd., Lipu, 546600 Guangxi China

**Keywords:** Drug discovery, Molecular biology

## Abstract

Intramuscular injection of anemoside B4 (AB4) has a superior therapeutic effect on clinical mastitis in lactating cows. Here, we explored AB4’s effect on milk whey in clinical mastitis-affected cows using proteomics. Among fifty clinical mastitis cows received AB4 administration (0.05 ml/kg/day, for 7 days), twelve healed cows were selected and marked as group T. Twelve clinically heathy cows received the same dose of saline for 7 days, marked as group C. Collected milk whey of group T before and after AB4 administration marked as T1 and T2, respectively. The milk whey of group C after saline injection marked as C1. Milk whey protein changes were detected using tandem mass tag-based quantitative proteomic. We identified 872 quantifiable proteins in the samples. Among them, 511 proteins between T1 and C1, and 361 proteins between T2 and T1 were significantly altered. T1 than C1 had significantly more proteins associated with inflammatory damage and trans-endothelial migration of leukocytes, whereas these proteins were reduced in T2 treated with AB4. Compared with C, proteins associated with fibrin clot degradation and complement system activation were downregulated in T1 but upregulated in T2. In summary, AB4 can exert its therapeutic effect on clinical mastitis in cows mainly by reducing inflammatory damage, activating the complement system, inhibiting trans-endothelial migration of leukocytes, and promoting degradation of milk fibrin clots.

## Introduction

*Pulsatilla chinensis* (Bunge) Regel, (a synonym of *Anemone chinensis* Bunge) belongs to the family of Ranunculaceae, being used as a kind of medical herb for a long history. In traditional Chinese medicine (TCM), *P. chinensis* is used to clear heat in body and detoxify, to cool blood and stop dysentery such as bacterial dysentery, amebic dysentery, especially good at clearing damp-heat of gastrointestinal tract and blood-heat toxin, in addition, it was reported to treat malaria and relieve spasm and pain as well^1^. Modern pharmacological studies confirmed that the extracts or the active compounds, such as anemoside B4 (AB4), anemoside B5 (AB5), isolated from *P. chinensis* played important roles in anti-inflammatory, anti-microbial and antiviral^2,3^. AB4 can inhibit the increase of TNF-α and E-selectin secretion from RIMECs caused by LPS with IL-6 unchanged, inhibits both LPS and concanavalin A-induced splenic lymphocyte proliferation, and reduces LPS-induced NF-κB activation and IL-6 production^4,5^. The in vivo anti-inflammatory activities of AB4 assessed through the intraperitoneal injection of 2 mg/kg AB4 in mice showed protection against LPS-induced acute lung injury, accompanied by reduction of IL-6, neutrophil infiltration, and NF-κB activation^6^. AB4 (12.5–50 mg/kg) significantly suppresses xylene-induced mouse ear edema and ameliorated LPS-induced kidney and lung inflammation damage, inhibiting pro-inflammatory response by NF-κB pathway in mice^4^. Findings from these in vitro and in vivo studies demonstrate the anti-inflammatory and immunomodulatory effects of AB4, highlighting its utility as a potential therapy for inflammation and immune response-related conditions, which is clearly related to its’ traditional use.

Mastitis, with an incidence and prevalence ranging from 20% to 60 in dairy herds, respectively is the most common disease in dairy cows^7^. It results in considerable monetary losses to the dairy industry due to decreased milk yield and quality, reduced reproductive performance, treatment, and premature culling^7–9^. Mastitis is the inflammation of the mammary gland, typically arising due to intramammary infections, broadly classified into subclinical (SCM) and clinical mastitis (CM) based on the severity of infection^10^. CM displays symptoms such as fever, depression, and anorexia and is usually associated with visible local and systemic signs of inflammation, marked by changes in milk, such as the appearance of clots, flakes, or watery texture^11,12^. CM is mainly caused by infection with pathogenic microorganisms such as *Escherichia coli*, *Streptococcus agalactiae*, and *Staphylococcus aureus*. *E. coli* releases bacterial cell wall components, such as lipopolysaccharide (LPS), which induces markedly increased local inflammatory mediators with a strong systemic acute phase response^13^. Activation of TLR4-dependent signaling pathways by LPS results in either the production of proinflammatory factors or initiation of endothelial apoptosis. Blocking TLR4 activation may inhibit the inflammatory response caused by LPS^14^. The *S. agalactiae* infection may affect cell proliferation and milk composition by reducing immune cell migration and inhibiting ATP synthesis and pathogen recognition pathways, thereby preventing immune response^7^. *S. aureus* is a gram-positive, opportunistic, and contagious bacterium that is one of the most prevalent causes of mastitis in cattle^15^. *S. aureus*-induced mastitis is difficult to eradicate with available therapies and is prone to persistent or recurrent infections due to the mechanisms that escape the attack from the host’s immune^16^. The desire to eradicate pathogens and assist the immune response established the routine use of intramammary antibiotics for the treatment of CM^17^. However, the extensive use of antibiotics has led to the evolution of antibiotic resistance in many bacterial species^18^. Therefore, government regulations have encouraged reduced antibiotic usage.

Previous studies indicate that intramuscular AB4 injection significantly attenuates clinical signs and manifestations in dairy cows with mastitis^19^. Here, we analyzed milk whey from dairy cows intramuscularly injected with AB4 after natural infection of CM using a tandem mass tag (TMT) proteomic approach and aimed to detect target proteins and molecular pathways associated with the therapeutic effect of AB4.

## Material and methods

### ARRIVE statement

This study was carried out in compliance with the ARRIVE guidelines.

### Ethics approval and consent to participate

All experiments were performed in accordance with Ethics Statement Approval of Institute Animal Ethics (IAEC) committee guidelines and reviewed by the Institutional Animal Care and Use Committee of Sichuan Agricultural University (DYY-S20174610).

### Animal and treatment

Fifty natural infected CM Chinese Holstein cows, identified and confirmed by a veterinary surgeon according to the criteria of Turk^11^, with Somatic cell count (an increased SCC results from an inflammatory process due to the presence of an intramammary infection. The SCC of milk sample is 500,000 cells/ml or more, which usually indicates breast infection udder infection.) above 500,000 cells/mL, positive California mastitis test results (CMT reagent is graded a negative, trace, + 1, + 2, and + 3. The greater the degree of viscosity, the more cellular the milk and possibly the greater the degree of inflammation), clinical signs of mastitis (included changes in milk appearance (flakes and clots in milk) and different stages of udder inflammation (hyperemia, edema, pain, udder enlargement, and elevated udder temperature), were recruited to the study from a dairy farm in the region of Southern Sichuan Province. All CM cows received an intramuscular infusion of AB4 (Guangxi innovates medical technology Co., Ltd. Lipu, Guangxi, China) (0.05 mL/kg, once daily) for 7 days. During this period, clinical symptoms and milk traits were observed every day to judge the therapeutic effect. CM cows without clinical symptoms, such as change in the appearance of milk (color, viscosity, consistency; i.e., flaky sediments, watery appearance, discoloration) and local clinical signs of inflammation of the udder parenchyma (i.e., swelling, heat, pain, redness), were assessed as clinically cured. Among the clinical cured cows, twelve clinical cured cows, at the 2nd–3rd lactation stage, weighted 565–659 kg and aged 3–4 years, were selected and marked as group T. Before (T1) and after (T2) AB4 injection, 10 mL milk samples were collected from groups T. Twelve health cows with same lactation stage, body weight and age, received an intramuscular injection of same dose saline for 7 days, marked as group C. Collected 10 mL milk samples of group C after saline injection (C1). There are 3 groups in this study. Each group has 3 biological replicates, which consists of 4 milk samples. Sample details and TMT tag information are shown in Table S1. The detailed information on the feed of the animals including proximate analysis as feed were provided in Table S2.

### Sample collection and milk whey preparation

Scrub teats with a pad soaked in 70% alcohol (Chengdu Kolon Chemicals Co., CN) and dried; massage the udder and discard the first three streams of milk from the teat before sampling. Collected milk samples was centrifuged at 10,000×*g* for 10 min to remove the cream layer (MTX 150 centrifuge, Thermo Fisher Scientific, USA). Then, CaCl_2_ (Chengdu Kolon Chemicals Co., CN) was added to a final concentration of 60 mM. The skim milk was acidified at pH 4.6 with the addition of 10% hydrochloric acid (Chengdu Kolon Chemicals Co., CN) to precipitate casein. The whey protein was centrifuged at 189,000×*g* for 60 min. The supernatant was collected for further TMT analysis. Three sample pools of each group were prepared by mixing milk whey from the four dairy cows. Meanwhile, SCC and inflammatory factors (IL-1β, IL-6, IL-8, IL-10, TNF-α) in whey were detected by SCC counter (Zhong Lao Technologies Ltd., CN) and ELISA kit (Nanjing Jiancheng Bioengineering Institution., CN) to evaluate the effectiveness of AB4 treatment.

### Protein digestion and TMT labeling

Sample proteins were extracted using the SDT method (4% w/v SDS, 100 mM Tris/HCl pH 7.6, 0.1 M DTT)^20^. Protein concentration was determined using the BCA kit (Thermo Fisher Scientific, USA). Proteins were digested using filter-aided proteome preparation (FASP) with trypsin (Solarbio, CN) hydrolysis^20^. The peptides derived from pooled samples were labeled using a TMT kit (Thermo Fisher Scientific, USA) according the manufacturer’s instruction. The samples were labeled as 128 (C1), 129 (T1) and 130 (T2). The TMT labelling efficiency was 100%.

### Fractionation of labeled peptides using high pH reversed-phase column

A C18 fractionation column (Thermo Scientific Acclaim PepMap100, 100 μm × 2 cm, nanoViper C18, USA) was preequilibrated with 0.1% (v/v) trifluoroacetic acid (Solarbio, CN) and eluted using an acetonitrile (containing 0.1% trifluoroacetic acid) gradient. Loaded the labeled peptides onto the analytical column (Thermo scientific EASY column, 10 cm, ID75μm, 3 μm, C18-A2, USA), followed by the addition of deionized water. Centrifuged the mixture at a low speed to desalt (Micro 17, Thermo Fisher Scientific, USA). Eluted the peptide from the C18 column using a gradient of acetonitrile. Digested peptide samples were dried in vacuum freeze dryer (LyoQest-55, Telstar, Spain) and reconstituted in trifluoroacetic (Chengdu Kolon Chemicals Co., CN). Detected the peptide concentration at OD280 (Nano Drop One^C^, Thermo Fisher Scientific, USA).

### High-resolution liquid chromatography-tandem mass spectrometry (LC–MS/MS) analysis

We run 80,126 spectra on the analytical column and get 11,235 TMT-labeled peptides. Then, LC–MS/MS analysis of TMT-labeled peptides was carried out using an HPLC system (Thermo Fisher Scientific, USA) coupled to an Easy nLC system (Thermo Fisher Scientific, USA). TMT-labeled peptides were dissolved in 0.1% formic acid (V/V) acetonitrile solution (Chengdu Kolon Chemicals Co., CN), loaded onto a C18 column (Thermo Scientific Acclaim PepMap100, 100 μm × 2 cm, nanoViper C18, USA), and separated on an analytical column (Thermo Fisher Scientific EASY column, 10 cm, ID75μm, 3 μm, C18-A2, USA) at a flow rate of 300 μL/min.

The MS was operated in positive ion mode using Q-Exactive. Full-scan MS spectra were acquired in the range from 300 to 1800 m/z. The MS1 resolution was 70,000 at 200-m/z. The automated gain control (AGC) target was 1e6. The maximum IT was 50 ms. The dynamic exclusion was 60 s. The 20 strongest signals of the parent ions were selected for secondary fragmentation. The MS2 activation type was high-energy collision dissociation (HCD). The isolation window was 2 m/z. The MS2 resolution was 35,000 at 100 m/z (TMT 10-plex).The normalized collision energy was 30 eV.

Analyzed the RAW data of the MS results by Mascot 2.2 and Proteome Discoverer 1.4. The relevant parameters and descriptions were as follows: enzyme, trypsin; max missed cleavages, 2; fixed modifications, Carbamidomethyl (C), TMT 10plex (N-term),TMT 10 plex (K); peptide mass tolerance, ± 20 ppm; fragment mass tolerance, 0.1 Da; peptide FDR, ≤ 0.01. Proteins’ GenInfo identifier (GI) accession numbers were converted into official gene symbol by uniport (https://www.uniprot.org/)-Bos-taurus-20191014.

### Data and statistical analysis

Gene ontology (GO database, http://www.geneontology.org) and Kyoto Encyclopedia of Genes and Genomes (KEGG database, http://www.genome.jp/kegg/)^21^ pathway analysis was performed using Blast2Go (https://www.blast2go.com/). Fisher’s exact test was used to analyze the GO and KEGG functional enrichments. Functional network analysis was performed using STRING (http://string-db.org/) and Cytoscape platform version 3.7.2 (https://cytoscape.org) based on bos-taurus genes.

## Results

### Clinical cure rate of AB4 in CM

The overall clinical cure rate of AB4 in CM was 76% (38/50).

### Milk whey protein changes

**Figure 1:**
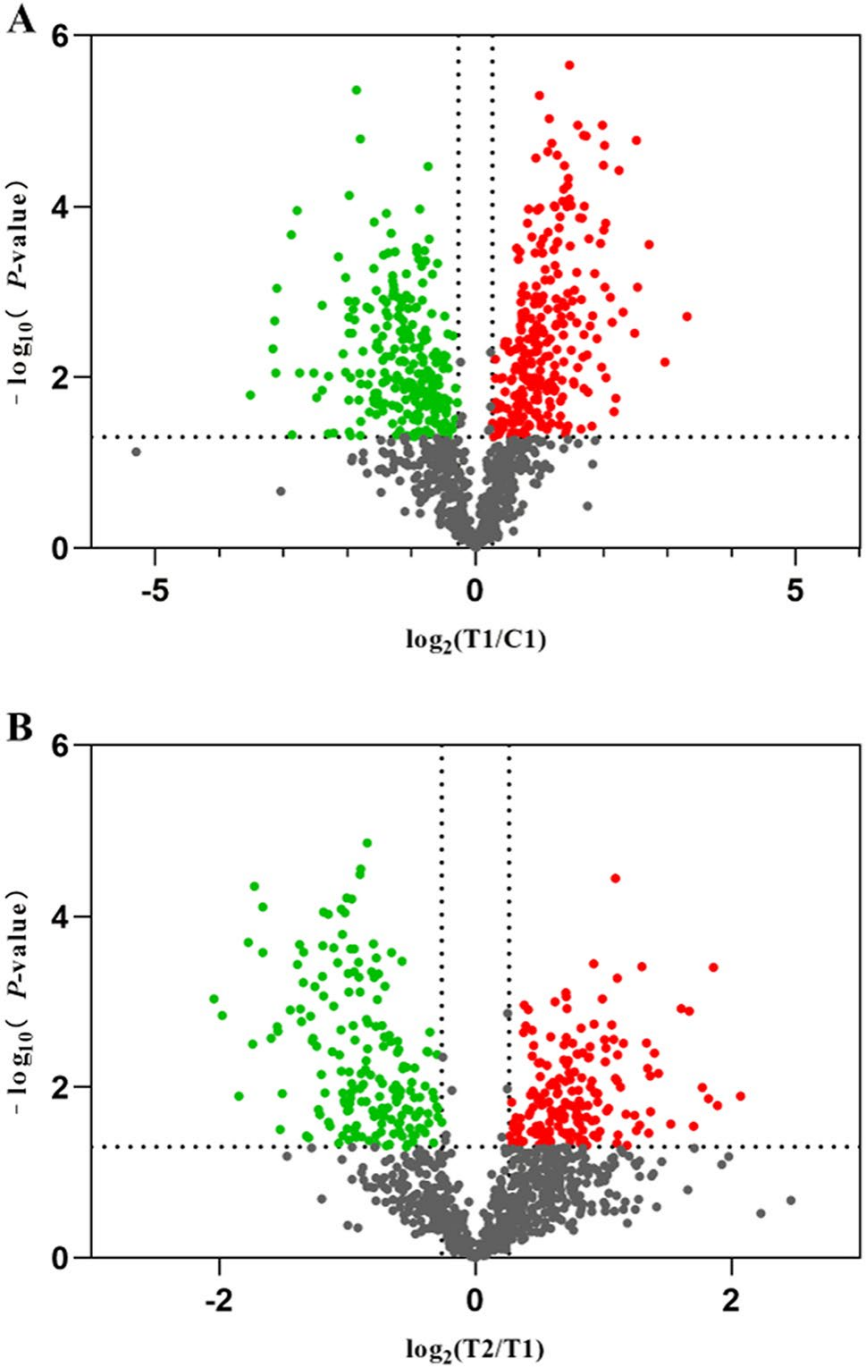
Volcano plot showing differentially abundant proteins between each group. (**A**) T1 vs C1; (**B**) T2 vs T1. The X-axis is the log of the fold-change with 2 as the base, and the Y-axis is the negative log of the p-value with 10 as the base. Gray dots show no significant difference in protein; red dots show significantly upregulated proteins, and green dots show significantly downregulated proteins.

We identified 1583 quantifiable proteins using a TMT-based quantitative proteomics approach according to the set criteria (one unique peptide, FDR ≤ 1%). Supplemental Table  S1 provides a complete list of the identified proteins. The differentially expressed proteins (DEPs) between each group were defined based on a 1.2-fold change threshold (with a fold change > 1.2 and < 0.83, *P* < 0.05) according to mass spectrum data. Supplemental Table S2 provided a full list of the DEPs. A total of 511 DEPs (268 upregulated and 243 downregulated) were detected between T1 and C1 (Fig. 1A). The results showed that 361 DEPs were identified between T2 and T1, 177 proteins were upregulated, and 184 proteins were downregulated (Fig. 1B).

### GO analyses

**Figure 2:**
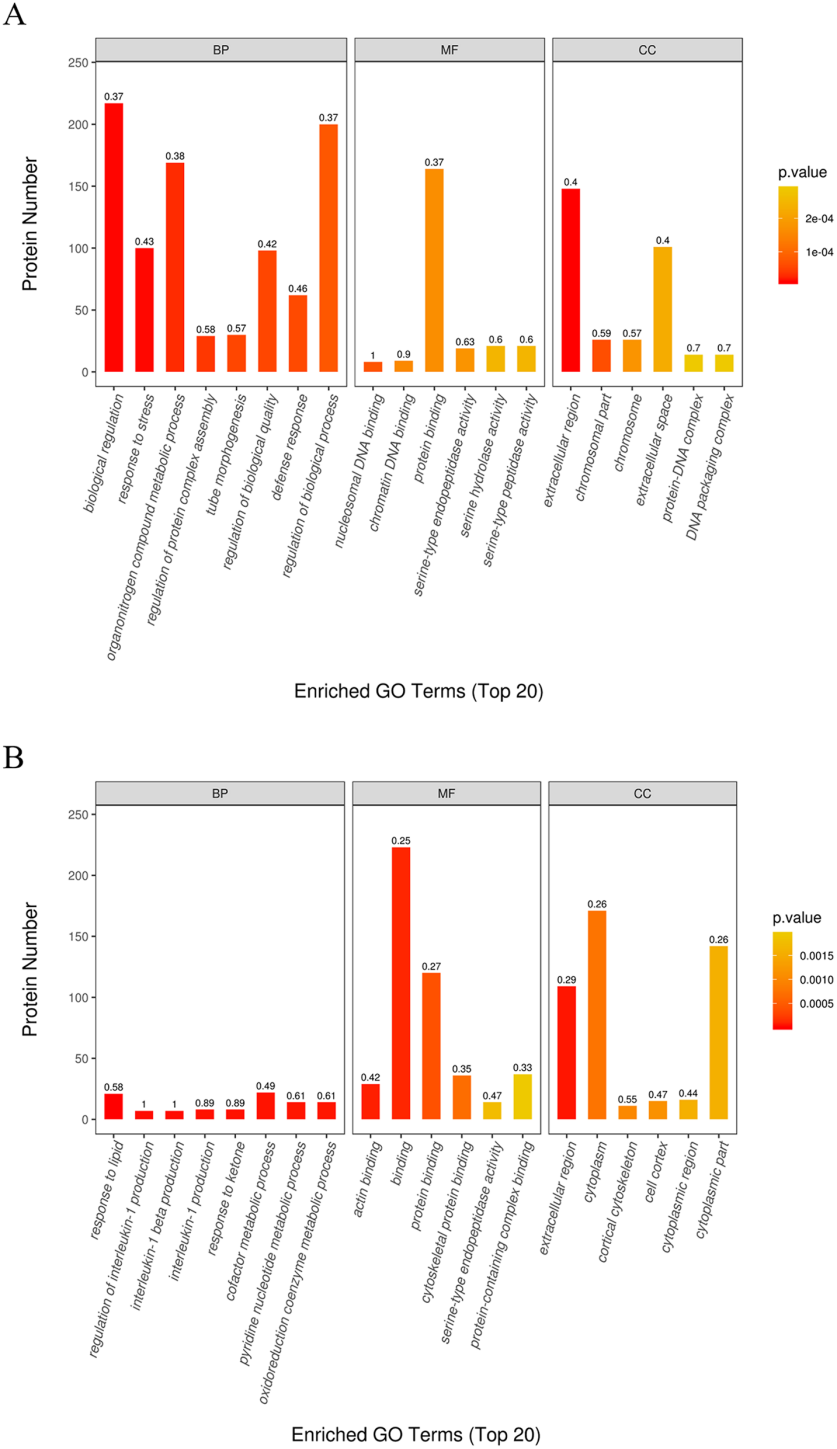
GO enrichment analysis of DEPs. Top 20 enriched GO terms of T1/C1 (**A**) and T2/T1 (**B**). The shade of box colors indicated the size of the *P*-value, the box height reflects the number of proteins involved in the GO terms, and the front on the box top was an enriched factor of this term.

We performed statistics on the distribution of DEPs using GO secondary annotation classification, including three major classes: biological processes (BP), cellular components (CC), and molecular functions. The top 20 enriched GO terms of T1/C1 and T2/T1 are shown in Fig. 2. Among the DEPs of T1/C1, BP included biological regulation (protein number = 217, *P* value = 0.37), stress response (protein number = 100, *P* value = 0.43), organonitrogen compound metabolic process (protein number = 169, *P* value = 0.38), etc. MF included nucleosomal DNA binding (protein number = 8, *P* value = 1.00), chromatin DNA binding (protein number = 9, *P* value = 0.90), and protein binding (protein number = 164, *P* value = 0.37). CC included the Golgi membrane (protein number = 12, *P* value = 0.50), nuclear chromosome (protein number = 11, *P* value = 0.58), chromatin (protein number = 20, *P* value = 0.59), etc. (Fig. 2A, Supplemental Table S3). Among the DEPs of T2/T1, BP included response to lipid (protein number = 21, *P* value = 0.58), interleukin-1 beta production (protein number = 7, *P* value = 1.00), regulation of interleukin-1 production (protein number = 7, *P* value = 1.00), etc. MF included serine-type endopeptidase activity (protein number = 14, *P* value = 0.47), cytoskeletal protein binding (protein number = 36, *P* value = 0.35), and transforming growth factor beta receptor binding (protein number = 4, *P* value = 1.00). CC included the extracellular region (protein number = 109, *P* value = 0.29), cytoplasm (protein number = 171, *P* value = 0.26), and the cortical cytoskeleton (protein number = 11, *P* value = 0.55) (Fig. 2B, Supplemental Table S4).

### KEGG analyses

**Figure 3:**
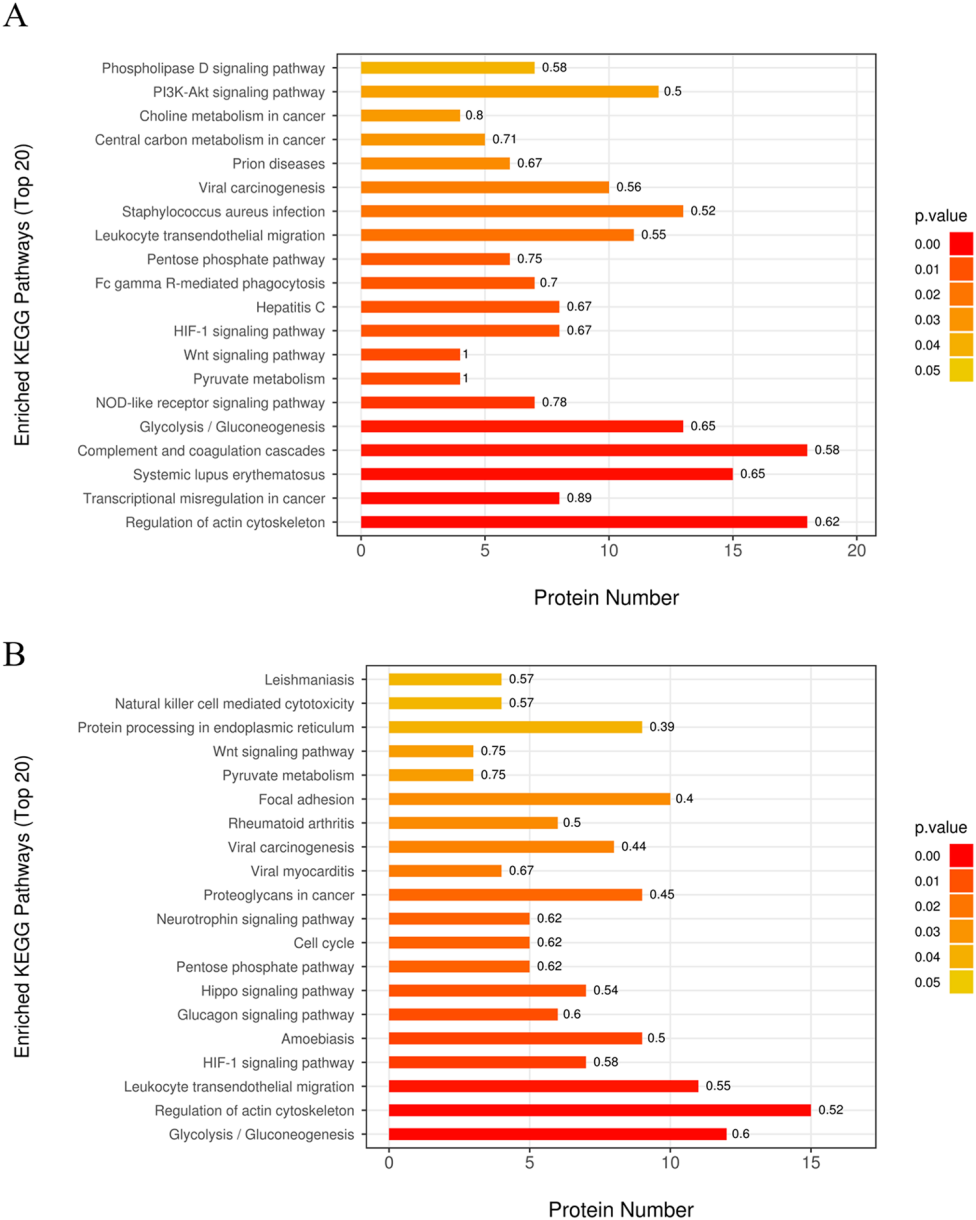
KEGG enrichment analysis of DEPs. Top 20 enriched KEGG pathways of T1/C1 (**A**) and T2/T1 (**B**). The shade of box colors indicated the size of the *P*-value, the box height reflects the number of proteins involved in the GO terms, and the front on the box top was an enriched factor of this term.

KEGG enrichment analysis results are shown in Supplemental Table S5. DEPs of T1/C1 enriched in 21 pathways and the top 20 are shown in Fig. 3B: complement and coagulation cascades, pyruvate metabolism, leukocyte transendothelial migration, regulation of actin cytoskeleton, etc. Additionally, DEPs of T2/T1 were enriched in 25 pathways (Fig. 3) and the top 20 pathways included focal adhesion, leukocyte transendothelial migration, regulation of actin cytoskeleton, etc. There were 10 common enriched pathways of T1/C1 and T2/T1: pyruvate metabolism, Wnt signaling pathway, pentose phosphate pathway, glycolysis/gluconeogenesis, HIF-1 signaling pathway, central carbon metabolism in cancer, leukocyte transendothelial migration, regulation of actin cytoskeleton, Fc gamma R-mediated phagocytosis, and viral carcinogenesis. Besides complement and coagulation cascades had 18 proteins in T1/C1 and eight proteins in T2/T1. In summary, the above mentioned 11 pathways were considered to have a potential role in AB4 (Table 1).

**Table 1:** Eleven potential target molecular pathways of AB4.

Map ID	Map name	Test sequence number	
		T1/C1	T2/C1
bta04610	Complement and coagulation cascades	18	8
bta00620	Pyruvate metabolism	4	3
bta04310	Wnt signaling pathway	4	3
bta00030	Pentose phosphate pathway	6	5
bta00010	Glycolysis/gluconeogenesis	13	12
bta04066	HIF-1 signaling pathway	8	7
bta05230	Central carbon metabolism in cancer	5	4
bta04670	Leukocyte transendothelial migration	11	11
bta04810	Regulation of actin cytoskeleton	18	15
bta04666	Fc gamma R-mediated phagocytosis	7	5
bta05203	Viral carcinogenesis	10	8

### Target proteins analysis

**Figure 4:**
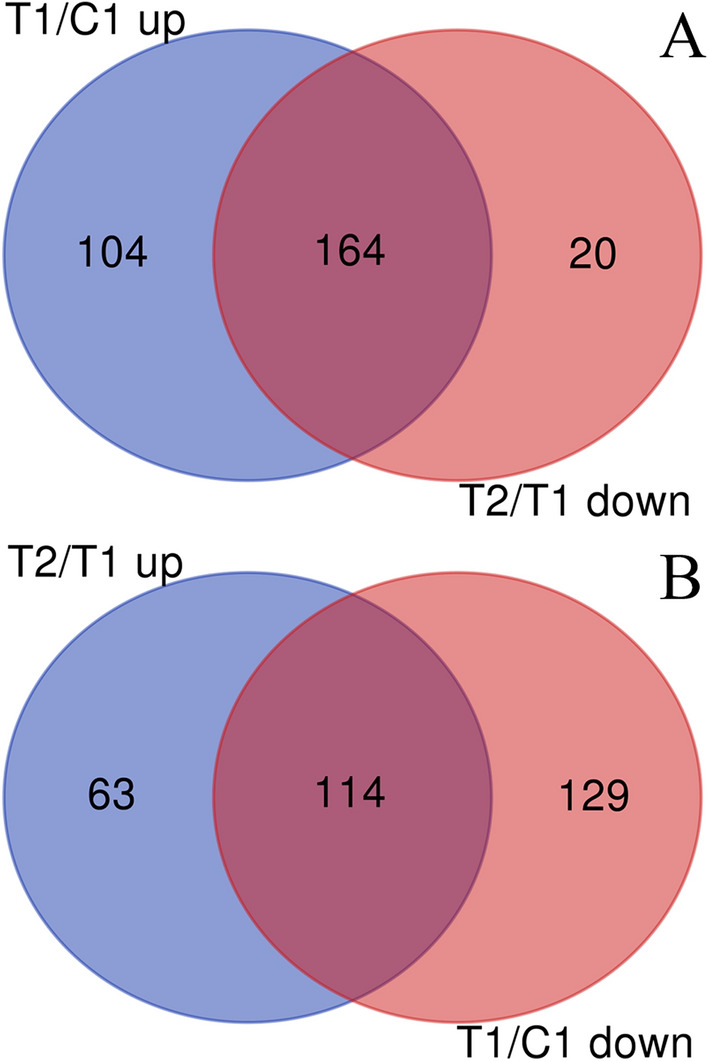
Venn diagram analysis. Upregulated proteins of T1/C1 and downregulated proteins of T2/C1 (**A**); upregulated proteins of T2/T1 and downregulated proteins of T1/C1 (**B**).

**Figure 5:**
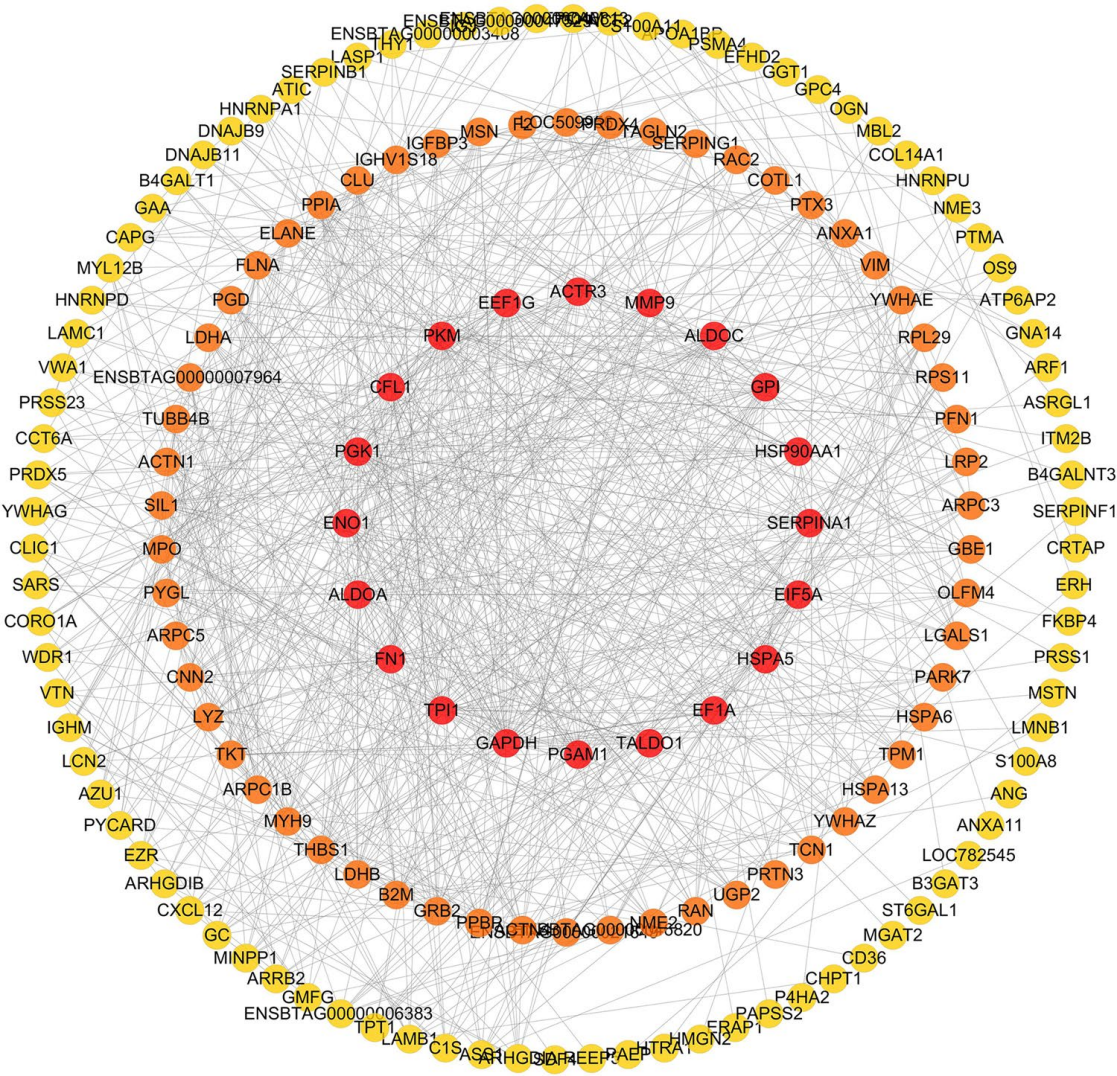
Protein–protein interaction (PPI) network. The yellow nodes represent the degree range of 1 to 10; The orange nodes represent the degree range of 10 to 20; The red nodes represent the degree above 20.

Among the 268 upregulated proteins in dairy cows with CM (T1/C1), 164 were downregulated after AB4 treatment (Fig. 4A). Additionally, 243 proteins were downregulated in cows with CM, 114 of which were upregulated following AB4 administration (Fig. 4B). These 274 proteins were potential targets for AB4. The PPI network revealed potential connections between the targets. Following the removal of free proteins, the PPI network contained 167 nodes and 5116 edges, with an average node degree of 10.07. The color of a node reflected the degree of importance. The larger the degree, the more important the node was in the network, suggesting that it may be a key target of B4 treatment. According to the degree value, hub proteins were defined as proteins with a degree greater than 20, as shown in Fig. 5 (red nodes).

## Discussion

We previously demonstrated that an IM injection of AB4 was therapeutically effective in naturally infected clinic mastitis in dairy cows^19^. Here, we identified 872 proteins in milk whey from dairy cows using the TMT proteomic approach. Among these proteins, 361 proteins significantly changed after IM injection of B4 in dairy cows with CM. These changes in proteins might be caused by the anti-inflammatory effect of AB4 on mastitis in dairy cows.

CM is caused by the invasion of pathogens. Gram-negative bacteria, such as *E. coli*, invade the mammary gland with LPS release, and the TLR4/nuclear factor-κB (NF-κB) signaling pathway is activated to produce pro-inflammatory factors and APPs, and cause mastitis^22^. *S. aureus* colonizes the mammary gland, adheres to the host epithelial cells and their extracellular matrix, synthesizes and secretes factors that allow the invasion, penetration, and destruction of the mammary tissue, including several exotoxins (hemolysins and leukocidins) and various hydrolytic enzymes such as proteases, coagulase, lipases, and hyaluronidases^23,24^. Additionally, *S. aureus* can escape, and also modulate the host immune system by producing a range of factors such as *S. aureus* superantigen toxins, protein A, and polysaccharide capsule^25^.

Turk et al. (2021) found that proteins involved in host defense such as α2- macroglobulin (A2M), α1-microglobulin/bikunin protein (AMBP), proteins involved in transport such as apolipoproteins AII and F (APOA2, APOF), and retinol-binding protein 4 (RBP4), are increased in milk but reduced in the serum of mastitis cows^26^. However, we found that APOA1, APOA4, APOE, and RBP4 were downregulated during clinical mastitis. These proteins were transferred from the blood to the pathogens causing mastitis. When the consumption of these proteins was greater than the rate of transfer from the blood, this may have caused conflicting results. This indicated that changes in the abundance levels of these proteins were not sensitive enough to act as biomarkers to predict infection of the mammary gland. Proteins involved in innate immunity and antimicrobial functions (e.g., serotransferrin, complement C3, fibrinogen gamma-B chain, and cathepsin B) and are associated with the immune response to pathogens (e.g., polymeric immunoglobulin receptor-like protein, MHC class I antigen, and beta-2-microglobulin) are abundantly expressed in whey from *S. aureus* mastitis milk^27^. HP, SAA1, DEFB10, and SERPINB3 are also observed to increase in milk from cows with mastitis^28^. Sudipa et al. Changes in the expression of HP and FN from Holstein Friesian correlated with disease progression, and angiogenin and cofilin-1 were upregulated while ubiquitin family members were downregulated during disease transition^29^. Proteins that stand out as logical candidates for further analyses include various APPs and vascular-derived proteins such as C3, C4, TF, ALB, TTR, FGA, and ITIH4 in mastitis whey of *E. coli* infected cows by 2-DE and label-free methods, respectively^30,31^. Host defense-related proteins such as HP, SERPINB1, SERPINB4, and ITIH4 were upregulated in our study, consistent with previous studies.

DEPs of *S. agalactiae*-induced mastitis are mostly enriched in complement and coagulation cascades, glycolysis/gluconeogenesis, pentose phosphate pathway, purine metabolism, NOD-like receptor signaling pathway, inflammatory mediator regulation of TRP channels, bacterial invasion of epithelial cells, chemokine signaling pathway proteasome, cell adhesion molecules (CAMs), and ribosomes^7,32^. *S. aureus-infected* mastitis also detected complement and coagulation cascades, pentose phosphate pathway, Fc gamma R-mediated phagocytosis pathway, leukocyte transendothelial migration pathway, acute phase response signaling, LXR/RXR activation, antigen processing and presentation pathway, and ECM–receptor interaction pathway^27,33,34^. *E. coli*-induced mastitis also found complement and coagulation cascades pathway, glycolysis/gluconeogenesis, pentose phosphate pathway, acute phase response signaling, lysosome, cytokine-cytokine receptor interaction, and cell adhesion molecules.

The top canonical pathways detected in *E. coli* were complement and coagulation cascades, glycolysis/gluconeogenesis, pentose phosphate pathway, leukocyte transendothelial migration^33,35^. Most of the AB4-targeted pathways identified here were consistent with the above pathways, including glycolysis/gluconeogenesis, pentose phosphate pathway, leukocyte transendothelial migration pathway, Fc gamma R-mediated phagocytosis, regulation of actin cytoskeleton, and complement and coagulation cascades.

Glycolysis is the process of converting glucose into pyruvate and generating small amounts of ATP (energy) and NADH (reducing power)^36^. Gluconeogenesis is a synthesis pathway of glucose from no carbohydrate precursors, and it is essentially a reversal of glycolysis with minor variations in alternative paths^37^. When mastitis developed (T1/C1), GPI, TPI1, GAPDH, PGK1, PGAM1, ENO1, and PKM in the glycolysis/gluconeogenesis pathway were upregulated, which promoted pyruvate synthesis with large amounts of ATP production. ATP is present in inflamed tissues in vivo at extracellular concentrations sufficient for P2 receptor activation, promoting leukocyte recruitment and NALP3-inflammasome activation via P2X7^38^. Lowering extracellular ATP levels in inflamed tissues can inhibit inflammation. Following AB4 treatment, GPI, TPI1, GAPDH, PGK1, PGAM1, ENO1, and PKM were downregulated, stimulating ATP breakdown to reduce inflammatory damage. LDHA and LDHB which were downregulated promote anaerobic metabolism of pyruvate with l-lactate and NADPH production^39^. This results in less energy for the cow and is not conducive to fighting inflammation. LDHA and LDHB were upregulated with AB4 treatment, which enhanced the aerobic metabolism of pyruvate to provide energy for fighting against inflammation. In the meantime, GPI, G6PD, PGD, and TALDDO1 in the pentose phosphate pathway was upregulated in CM cows, leading to bulk NADPH production. Activated in nature by microbes and microbial-derived products, the phagocyte NADPH oxidase rapidly assembles and generates reactive oxidant intermediates (ROIs) in response to infectious threats^40^. NADPH oxidase plays a key role in modulating inflammation and injury, distinct from its antimicrobial function^40^. However, ROIs can directly injure cells by damaging DNA, proteins, and lipids^41^. GPI, G6PD, PGD, and TALDDO1 downregulation with AB4 can prevent oxidative damage by excessive NADPH.

Leukocyte migration from the blood into the sites of infection is a vital immune surveillance strategy^42^. In cows with CM, Rac2 upregulation promotes leukocyte motility^43^; RhoA and MLC upregulation indirectly promotes tail retraction^44^; ERM, actin, and α-actin upregulation indirectly regulates the docking structure^45^, and CLDN8 downregulation promotes leukocyte transendothelial migration. This is also responsible for the high SCC in mastitis milk. After AB4 treatment, Rac2, MLC, ERM, actin, and α-actin were downregulated, and leukocyte migration was inhibited. ITGB2 binds to JAM1, JAM3, and ICAM3, promoting transendothelial migration of neutrophils and T cells, and phagocytosis of apoptotic neutrophils by macrophages, respectively^46,47^. ITGB2 was downregulated by AB4. These results indicate that AB4 has a great potential to reduce SCC.

Phagocytosis plays an essential role in host defense mechanisms through the uptake and destruction of infectious pathogens^48^. After opsonization with antibodies (IgG), foreign extracellular materials are recognized by Fc gamma receptors. IgG is downregulated in mastitis cows, reducing the ability to recognize antigens, be caused by a vast array of immune evasion mechanisms of *S. aureus*. CFL1 plays a role in the regulation of cell morphology and cytoskeletal organization in epithelial cells^49^. The Arp2/3 complex mediates the formation of branched actin networks in the cytoplasm, providing a force for cell motility^50^. CFL1, ARPC4, and ARPC3 are upregulated during cow mastitis, promoting macrophage, neutrophil, and monocyte motility to destroy infectious pathogens^51,52^. Following AB4 treatment, IgG was upregulated, whereas CFL1, ARPC4, and ARPC3 were downregulated, indicating that invasive pathogens were effectively controlled.

The most common pathway of mastitis infection is the complement and coagulation cascade pathway, also the main pathway for the host to deal *with S. aureus*^34^*.* The complement system is a proteolytic cascade in blood plasma and a mediator of innate immunity, a nonspecific defense mechanism against pathogens. The main consequences of complement activation are the opsonization of pathogens, recruitment of inflammatory and immunocompetent cells, and direct killing of pathogens^53^. Pathogens have a greater effect on the blood-milk barrier, and consequently, a larger transfer of blood proteins to milk occurs in mastitis^54^. Together with fibrinogen alpha (FGA) and fibrinogen beta (FGB), polymerize to form an insoluble fibrin matrix^55^. FGA and FGB are upregulated with fibrin monomer production, further transfer to fibrin clots, causing deterioration in milk quality. Plasmin, which transfers fibrin clots to fibrin degradation products via the fibrinolytic system, reduced production due to the downregulation of PLG, HCII, F2, A1AT, and α2AP. C1q is the first subcomponent of the C1 complex in the classical pathway of complement activation^56^. Functions in the lectin pathway of complement play a key role in innate immunity by recognizing pathogens through patterns of sugar moieties and neutralizing them. The lectin pathway is triggered upon the binding of mannan-binding lectin (MBL) and ficolin to sugar moieties, leading to activation of the associated proteases MASP1 and MASP2^57^. Here, C1, MBL, MASP1, and MASP2 were downregulated in mastitis. The classical and lectin pathways of the complement cascade were inhibited, and AB4 treatment C1 and MBL were upregulated, activating the complement system. VTN and CLU protect cells against apoptosis and cytolysis by complement^58,59^. Bacterial invasion into the mammary gland induced VTN and CLU downregulation, promoting cell apoptosis and cytolysis. Both VTN and CLU were upregulated after AB4 treatment. The membrane attack complex (MAC) forms trans–plasma membrane channels on the surface of pathogenic bacteria, causing cell lysis and death; only five of the complement system proteins eventually formed MAC subunits: one unit each of complement C5b, C6, C7, and C8 and several units of complement C9^60^. C8A, C8B, C8G, and C9 were downregulated in mastitis, indicating that the host's ability to eliminate the bacteria is diminished. Following AB4 treatment, all proteins were restored to normal levels.

In summary, pathogenic bacterial infection upregulated PKM, LDHB, LDHA, ALDOA, PGD, GPI, and ALDOC, increased ATP production, which promotes leukocyte recruitment and NALP3-inflammasome activation, increases NADPH production, and promotes ROIs modulating inflammation and injury. These proteins were restored by AB4, which indicates that inflammation was inhibited. Invasion of pathogenic bacteria leads to transdermal migration of leukocytes into the mammary tissue, elevating somatic cells in the milk, increasing fibrinogen precursors, and decreasing plasmin production, resulting in fibrin clot deposition, eventually causing deterioration in milk quality. Bacterial infection of mammary tissue inhibits activation of the complement system, reduces MAC production, and protects pathogenic bacteria from killing through the complement immune system, thereby promoting apoptosis of mammary epithelial cells. Intramuscular infusion of AB4 can downregulate GPI, TPI1, GAPDH, PGK1, PGAM1, ENO1, PKM, GPI, G6PD, PGD, and TALDDO1 and restore LDHB and LDHA, reducing inflammatory damage caused by ATP and NADPH; downregulating Rac2, RhoA, MLC, ERM, actin, and α-actin ITGB2, inhibiting transendothelial migration of leukocytes, thereby reducing milk SCC; restoring PLG, FGA, FGB, reducing milk fibrin clots; upregulate C1, MBL, CLU, VTN, activating the complement system and reducing MAC, and directly inhibiting the invasion of pathogenic bacteria.

## Conclusions

We investigated the differences in the milk proteomes of mastitis-affected cows following intramuscular AB4 injection. Our results suggested that AB4 may treat CM through multiple pathways by easing inflammation, reducing SCC, decreasing milk clots, and eradicating pathogenic bacteria. These results contribute to a better understanding of the AB4 mechanism in CM, providing theoretical support for its clinical application. Application of AB4 can reduce antibiotic usage, therefore, economic losses because of antibiotic residues in milk can be avoided, bacterial resistance will be controlled, and environmental pollution caused by antibiotic misuse will be reduced. Future research confirming the target proteins identified in this study of AB4 on dairy cows with CM is needed.

## Supplementary Information

Supplementary Information 1.

Supplementary Table S1.

Supplementary Table S2.

Supplementary Table S3.

Supplementary Table S4.

Supplementary Table S5.

## Data Availability

Proteins’ GenInfo identifier (GI) accession numbers were converted into official gene symbol by uniport (https://www.uniprot.org/)-Bos-taurus-20191014. The full list of proteins identified in the milk whey samples are provided in the supplemental Table S1. DEPs of T1/C1 and T1/T2 are provided in supplemental Tables S2. Tables S3 and S4 show enriched GO terms of DEPs. Table S5 lists significantly enriched KEGG pathways. The datasets generated during the current study are available in the Mendeley repository, Effect of anemoside B4 on milk whey in clinical mastitis-affected cows elucidated using tandem mass tag (TMT)-based quantitative proteomics-Mendeley Data.

## References

[1] Zhao Z, Nian M, Qiao H, Li B, Zheng X (2021). *Pulsatilla chinensis*: A review of traditional uses, phytochemistry and pharmacology research progress. Arab. J. Chem..

[2] Li W (2014). Characterization of an antiproliferative exopolysaccharide (LHEPS-2) from *Lactobacillus helveticus* MB2-1. Carbohydr. Polym..

[3] Sun Y, Liu J, Yu H, Gong C (2010). Isolation and evaluation of immunological adjuvant activities of saponins from the roots of *Pulsatilla chinensis* with less adverse reactions. Int. Immunopharmacol..

[4] Kang N (2019). Anti-inflammatory and immune-modulatory properties of anemoside B4 isolated from *Pulsatilla chinensis* in vivo. Phytomedicine.

[5] Hu Y, Chen X, Duan H, Hu Y, Mu X (2009). Chinese herbal medicinal ingredients inhibit secretion of IL-6, IL-8, E-selectin and TXB2 in LPS-induced rat intestinal microvascular endothelial cells. Immunopharmacol. Immunotoxicol..

[6] Zhou M, Chen L, Hu H, Luo Y, Fang C (2019). NZ protective effect of Pulsatilla saponin B4 on lipopolysacchariide-induced acute lung injury. Tradit. Chin. Drug Res. Clin. Pharmacol..

[7] Tong J (2020). Proteomic analysis of bovine mammary epithelial cells after in vitro incubation with *S. agalactiae*: Potential biomarkers. Vet. Res..

[8] Seegers H, Fourichon C, Beaudeau F (2003). Production effects related to mastitis and mastitis economics in dairy cattle herds. Vet. Res..

[9] De Graves FJ, Fetrow J (1993). Economics of mastitis and mastitis control. Vet. Clin. North Am. Food Anim. Pract..

[10] Bathla S (2020). Tandem Mass Tag (TMT)-based quantitative proteomics reveals potential targets associated with onset of sub-clinical Mastitis in cows. Sci. Rep..

[11] Turk R (2021). Milk and serum proteomes in subclinical and clinical mastitis in Simmental cows. J. Proteomics.

[12] Wollowski L (2021). The value of the biomarkers cathelicidin, milk amyloid A, and haptoglobin to diagnose and classify clinical and subclinical mastitis. J. Dairy Sci..

[13] Yan B (2017). Palmatine inhibits TRIF-dependent NF-κB pathway against inflammation induced by LPS in goat endometrial epithelial cells. Int. Immunopharmacol..

[14] Hu G (2018). Cynatratoside-C from *Cynanchum atratum* displays anti-inflammatory effect via suppressing TLR4 mediated NF-κB and MAPK signaling pathways in LPS-induced mastitis in mice. Chem. Biol. Interact..

[15] Hw B, Yh S, Rn Z (2006). Invited review: The role of cow, pathogen, and treatment regimen in the therapeutic success of bovine *Staphylococcus aureus* mastitis. J. Dairy Sci..

[16] Jingjing W (2021). DNase I improves blood-milk barrier integrity and alleviates inflammation induced by *Staphylococcus aureus* during mastitis. Int. Immunopharmacol..

[17] Ruegg PL (2017). A 100-year review: Mastitis detection, management, and prevention. J. Dairy Sci..

[18] Zhu YZ (2018). Anti-BACE1 and antimicrobial activities of steroidal compounds isolated from marine *Urechis unicinctus*. Mar. Drugs.

[19] Qian B, You L, Zhang Y, Shen Y, Lv S, Xiao J, Su Z, Dong K, Pei M, Zuo C, Yang S, Feng Y (2021). Effects of pulsatilla saponin B4 on somatic cell counts, enzymes, inflammatory and antioxidant factors in milk of dairy cows with clinical mastitis. Acta Agric. Univ. Jiangxiensis.

[20] Wiśniewski JR, Zougman A, Nagaraj N, Mann M (2009). Universal sample preparation method for proteome analysis. Nat. Methods.

[21] Kanehisa M, Furumichi M, Sato Y, Ishiguro-Watanabe M, Tanabe M (2021). KEGG: Integrating viruses and cellular organisms. Nucleic Acids Res..

[22] Yang W (2008). Bovine TLR2 and TLR4 properly transduce signals from *Staphylococcus aureus* and *E. coli*, but *S. aureus* fails to both activate NF-κB in mammary epithelial cells and to quickly induce TNFα and interleukin-8 (CXCL8) expression in the udder. Mol. Immunol..

[23] Middleton JR, Luby CD, Adams DS (2009). Efficacy of vaccination against staphylococcal mastitis: A review and new data. Vet. Microbiol..

[24] Suriyaphol G, Sarikaputi M, Suriyaphol P (2009). Differential responses of cells from human skin keratinocyte and bovine mammary epithelium to attack by pore-forming *Staphylococcus aureus* α-toxin. Comp. Immunol. Microbiol. Infect. Dis..

[25] Wang SC (2009). Distribution of superantigenic toxin genes in *Staphylococcus aureus* isolates from milk samples of bovine subclinical mastitis cases in two major diary production regions of China. Vet. Microbiol..

[26] Turk R (2021). Milk and serum proteomes in subclinical and clinical mastitis in Simmental cows. J. Proteomics.

[27] Abdelmegid S, Kelton D, Caswell J, Kirby G (2020). Proteomic 2d-dige analysis of milk whey from dairy cows with *Staphylococcus aureus* mastitis reveals overexpression of host defense proteins. Microorganisms.

[28] Thomas FC (2016). Mastitomics, the integrated omics of bovine milk in an experimental model of *Streptococcus uberis* mastitis: 3. Untargeted metabolomics. Mol. Biosyst..

[29] Maity S, Das D, Ambatipudi K (2020). Quantitative alterations in bovine milk proteome from healthy, subclinical and clinical mastitis during *S. aureus* infection. J. Proteomics.

[30] Boehmer JL (2010). The proteomic advantage: Label-free quantification of proteins expressed in bovine milk during experimentally induced coliform mastitis. Vet. Immunol. Immunopathol..

[31] Boehmer JL, Bannerman DD, Shefcheck K, Ward JL (2008). Proteomic analysis of differentially expressed proteins in bovine milk during experimentally induced *Escherichia coli* mastitis. J. Dairy Sci..

[32] Zhang H (2018). Transcriptomics and iTRAQ-proteomics analyses of bovine mammary tissue with *Streptococcus agalactiae*-induced mastitis. J. Agric. Food Chem..

[33] Ibeagha-Awemu EM, Ibeagha AE, Messier S, Zhao X (2010). Proteomics, genomics, and pathway analyses of *Escherichia coli* and *Staphylococcus aureus* infected milk whey reveal molecular pathways and networks involved in mastitis. J. Proteome Res..

[34] Cai L (2020). *Staphylococcus aureus*-induced proteomic changes in the mammary tissue of rats: A TMT-based study. PLoS ONE.

[35] Yang Y (2014). Proteomics and pathway analysis of N-glycosylated mammary gland proteins in response to *Escherichia coli* mastitis in cattle. Vet. J..

[36] GöranRonquist K (2019). Extracellular vesicles and energy metabolism. Clin. Chim. Acta.

[37] Leithner K (2021). New roles for gluconeogenesis in vertebrates. Curr. Opin. Syst. Biol..

[38] Leo Bours MJ, Dagnelie PC, Giuliani AL, Wesselius A, Di Virgilio F (2011). P2 receptors and extracellular ATP: A novel homeostatic pathway in inflammation. Front. Biosci. Sch..

[39] Adams MJ (1973). Structure-function relationships in lactate dehydrogenase. Proc. Natl. Acad. Sci..

[40] Segal BH, Grimm MJ, Khan ANH, Han W, Blackwell TS (2012). Regulation of innate immunity by NADPH oxidase. Free Radic. Biol. Med..

[41] Lee K, Ahn J-H, Lee K-T, Jang DS, Choi J-H (2018). Deoxyschizandrin, isolated from schisandra berries, induces cell cycle arrest in ovarian cancer cells and inhibits the protumoural activation of tumour-associated macrophages. Nutrients.

[42] Yang H (2011). Insight into bacterial virulence mechanisms against host immune response via the yersinia pestis-human protein-protein interaction network. Infect. Immun..

[43] Deng Q, Yoo SK, Cavnar PJ, Green JM, Huttenlocher A (2011). Dual roles for Rac2 in neutrophil motility and active retention in zebrafish hematopoietic tissue. Dev. Cell.

[44] Al-Dimassi S (2016). Targeting the MAP kinase pathway in astrocytoma cells using a recombinant anthrax lethal toxin as a way to inhibit cell motility and invasion. Int. J. Oncol..

[45] Dominguez R, Holmes KC (2011). Actin structure and function. Annu. Rev. Biophys..

[46] Kristóf E (2013). Novel role of ICAM3 and LFA-1 in the clearance of apoptotic neutrophils by human macrophages. Apoptosis.

[47] Ostermann G, Weber KSC, Zernecke A, Schröder A, Weber C (2002). JAM-I is a ligand of the β2 integrin LFA-I involved in transendothelial migration of leukocytes. Nat. Immunol..

[48] Swanson JA, Hoppe AD (2004). The coordination of signaling during Fc receptor-mediated phagocytosis. J. Leukoc. Biol..

[49] Bai SW (2011). Identification and characterization of a set of conserved and new regulators of cytoskeletal organization, cell morphology and migration. BMC Biol..

[50] Welch MD, DePace AH, Verma S, Iwamatsu A, Mitchison TJ (1997). The human Arp2/3 complex is composed of evolutionarily conserved subunits and is localized to cellular regions of dynamic actin filament assembly. J. Cell Biol..

[51] Nakano K (2003). Cofilin phosphorylation and actin polymerization by NRK/NESK, a member of the germinal center kinase family. Exp. Cell Res..

[52] Insall R (2001). Dynamics of the dictyostelium Arp2/3 complex in endocytosis, cytokinesis, and chemotaxis. Cell Motil. Cytoskeleton.

[53] Bajic G, Degn SE, Thiel S, Andersen GR (2015). Complement activation, regulation, and molecular basis for complement-related diseases. EMBO J..

[54] Bannerman DD (2004). *Escherichia coli* and *Staphylococcus aureus* elicit differential innate immune responses following intramammary infection. Clin. Diagn. Lab. Immunol..

[55] Renaud L, da Silveira WA, Takamura N, Hardiman G, Feghali-Bostwick C (2020). Prominence of IL6, IGF, TLR, and bioenergetics pathway perturbation in lung tissues of scleroderma patients with pulmonary fibrosis. Front. Immunol..

[56] Kishore U, Reid KB (2000). C1q: Structure, function, and receptors. Immunopharmacology.

[57] Rooryck C (2011). Mutations in lectin complement pathway genes COLEC11 and MASP1 cause 3MC syndrome. Nat. Genet..

[58] Hong JY (2021). Clusterin deficiency exacerbates hyperoxia-induced acute lung injury. Cells.

[59] Sjölinder M (2012). The meningococcal adhesin NhhA provokes proinflammatory responses in macrophages via toll-like receptor 4-dependent and -independent pathways. Infect. Immun..

[60] Stillwell, W. Chapter 20 - Bioactive Lipids. *An Introd. to Biol. Membr. *2nd ed, 453–478 (2016).

