# Supplementary material for: Effect of anemoside B4 on milk whey in clinical mastitis-affected cows elucidated using tandem mass tag (TMT)-based quantitative proteomics
Source: Sci Rep. 2022 Nov 5;12:18829. doi: 10.1038/s41598-022-23749-x (PMC9637092; doi:10.1038/s41598-022-23749-x)
Supplement: Supplementary file 1 — Supplementary Information 1. [file 41598_2022_23749_MOESM1_ESM.docx]

**Table S1** **Sample details and TMT tag information**

| TMT tag | 128 | 129 | 130 |
| --- | --- | --- | --- |
| Sample name | C1-1 | T1-1 | T2-1 |
|  | C1-2 | T1-2 | T2-2 |
|  | C1-3 | T1-3 | T2-3 |

**Table S2. Feed formula and nutritional composition of lactating cattle.**

| Feedstuff | （Dry matter%） |
| --- | --- |
| Premixed feed | 49.34 |
| Steam flaking maize | 3.318 |
| Beet pulp | 2.647 |
| Cottonseed | 7.390 |
| Corn silage | 14.96 |
| Alfalfa | 13.88 |
| Oat grass | 8.484 |
| Nutritional ingredient | （Dry matter%） |
| Dry matter | 56.7 |
| Crude protein | 17.1 |
| Neutral detergent fiber | 33.9 |
| Starch | 23.8 |
| Ca | 0.94 |
| P | 0.45 |

**Table S3** **Detection results of whey protein in Dairy cows**

| Database | Spectra | Peptides | Unique peptides | Total proteins |
| --- | --- | --- | --- | --- |
| uniprot Bos taurus | 80126 | 11235 | 7450 | 1677 |

Note: Spectra means peptide spectrum match.

**Figure S1 The SDS-PAGE of pooled samples**

**
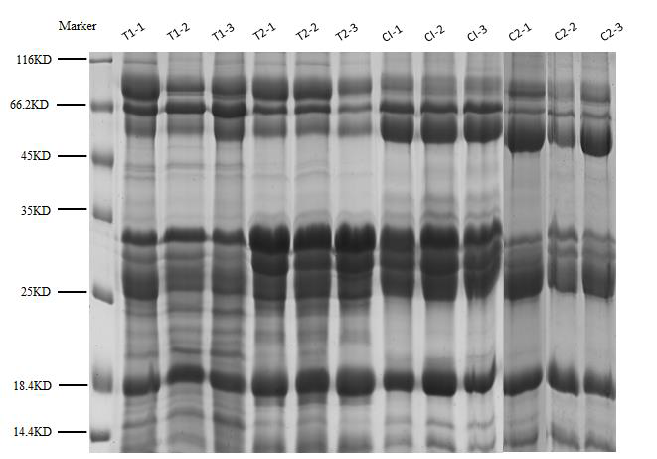
**

**Figure S2** **Representative LC-MS/MS Basepeak chromatograms**

**
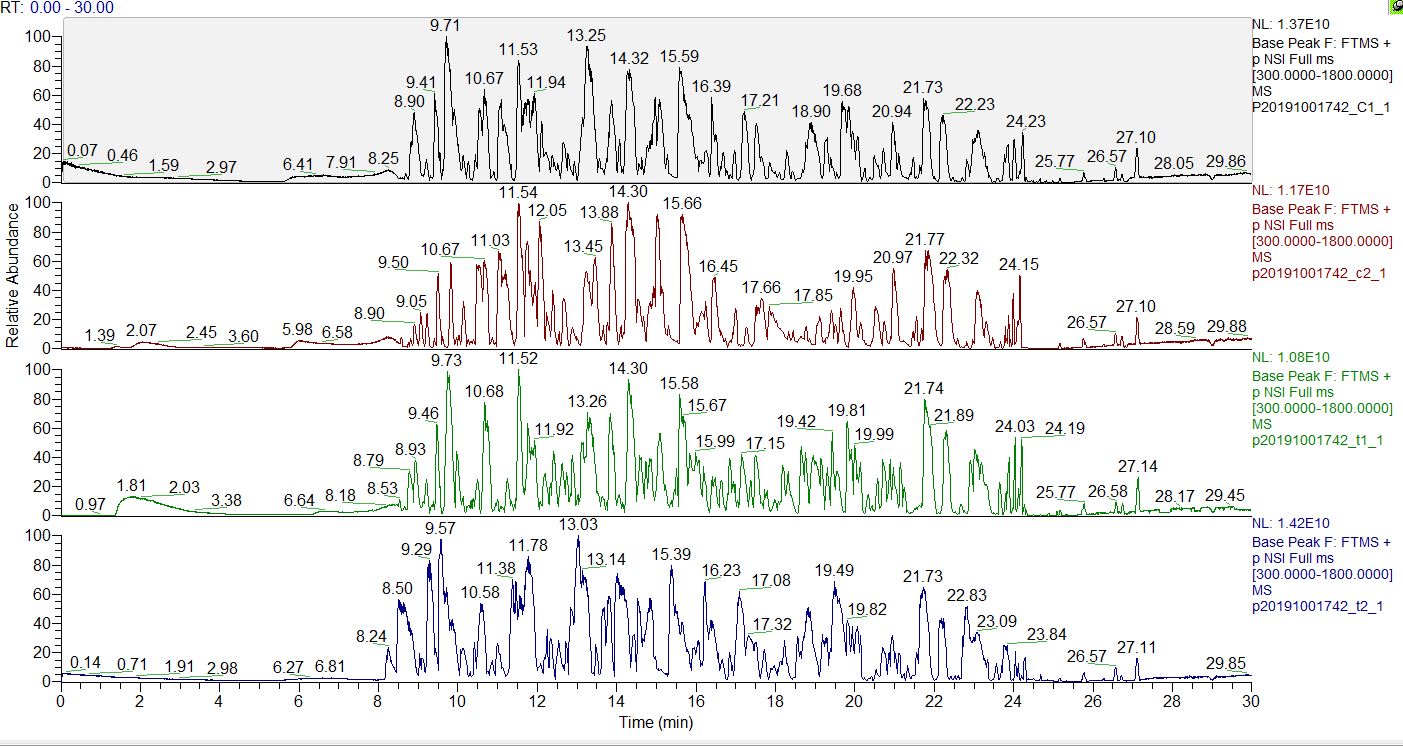
**

**Figure S3 Representative** **TOF/TOF mass spectra**
